# Controls of Evapotranspiration and CO_2_ Fluxes from Scots Pine by Surface Conductance and Abiotic Factors

**DOI:** 10.1371/journal.pone.0069027

**Published:** 2013-07-24

**Authors:** Tianshan Zha, Chunyi Li, Seppo Kellomäki, Heli Peltola, Kai-Yun Wang, Yuqing Zhang

**Affiliations:** 1 The School of Soil and Water Conservation, Beijing Forestry University, Beijing, China; 2 Faculty of Science and Forestry, University of Eastern Finland, Joensuu, Finland; 3 Urban Ecology and Restoration Key Laboratory, East China Normal University, Shanghai, China; The Ohio State University, United States of America

## Abstract

Evapotranspiration (*E*) and CO_2_ flux (*F_c_*) in the growing season of an unusual dry year were measured continuously over a Scots pine forest in eastern Finland, by eddy covariance techniques. The aims were to gain an understanding of their biological and environmental control processes. As a result, there were obvious diurnal and seasonal changes in *E, F_c_*, surface conductance (*g_c_*), and decoupling coefficient (*Ω*), showing similar trends to those in radiation (PAR) and vapour pressure deficit (*δ*). The maximum mean daily values (24-h average) for *E*, *F_c_*, *g_c_*, and *Ω* were 1.78 mmol m^−2^ s^−1^, −11.18 µmol m^−2^ s^−1^, 6.27 mm s^−1^, and 0.31, respectively, with seasonal averages of 0.71 mmol m^−2^ s^−1^, −4.61 µmol m^−2^ s^−1^, 3.3 mm s^−1^, and 0.16. *E* and *F_c_* were controlled by combined biological and environmental variables. There was curvilinear dependence of *E* on *g_c_* and *F_c_* on *g_c_*. Among the environmental variables, PAR was the most important factor having a positive linear relationship to *E* and curvilinear relationship to *F_c_*, while vapour pressure deficit was the most important environmental factor affecting *g_c_*. Water use efficiency was slightly higher in the dry season, with mean monthly values ranging from 6.67 to 7.48 μmol CO_2_ (mmol H_2_O)^−1^ and a seasonal average of 7.06 μmol CO_2_ (μmol H_2_O)^−1^. Low *Ω* and its close positive relationship with *g_c_* indicate that evapotranspiration was sensitive to surface conductance. Mid summer drought reduced surface conductance and decoupling coefficient, suggesting a more biotic control of evapotranspiration and a physiological acclimation to dry air. Surface conductance remained low and constant under dry condition, supporting that a constant value of surface constant can be used for modelling transpiration under drought condition.

## Introduction

The climate in the boreal zone has warmed already in the last century and is predicted to warm significantly further in this century [1]. Climate variability has the potential to affect carbon exchange, evapotranspiration, and other ecophysiological processes in forest ecosystems. Moreover, the extent of the boreal forests, their role in contemporary northern hemisphere climatology and the global carbon cycle, and their sensitivity to climate change are sufficient reasons for better understanding of boreal ecosystem-atmosphere interactions [2].

To obtain an understanding of environmental and biological controls of evapotranspiration and CO_2_ flux has been a central focus of climate change research for decades [3]. Evapotranspiration is an important process that is controlled by the interaction of a number of environmental factors (e.g., solar radiation, air temperature, vapour pressure deficit, and soil water content) and biological processes (e.g., leaf emergence, leaf development, and stomatal conductance) [4]–[9]. It has been found that biological control of evapotranspiration in a forest ecosystem is reflected in the form of changes in surface conductance [10], [11]. The transpiration from coniferous forests in the boreal region is largely controlled by canopy conductance [12], [13], because boreal forest canopies are aerodynamically rough and well ventilated, so that the effect of aerodynamic conductance is minimized.

CO_2_ flux is simultaneously regulated by canopy conductance, since both water vapour and CO_2_ pass through the stomata. Hence, stomatal conductance is not only the key to the assessment of transpiration and water balance, but also important for estimating carbon flux. The stomata are highly responsive to environmental variables such as high vapour pressure deficits [14], drying soils, and low light, so that all of these may act to regulate stomatal conductance.

Scots pine (*Pinus sylvestris* L.) is a major tree species in boreal areas, and its response to climate change and the roles it plays in carbon and water cycling are of great interest to ecologists. Understanding of the control of stomatal conductance over transpiration and photosynthesis at ecosystem level for Scots pine is limited relative to a large body of knowledge at leaf level [8]. As the spatial and temporal scale of our measurements and experiments has increased, more concerns have been focused on the understanding of how canopy conductance is involved in regulating carbon and water cycles on the ecosystem scale and over long periods of time [14]–[16]. Many previous reports have pointed to seasonal and interannual variations in the fluxes of carbon and water in forest ecosystems [3], [17], [18]. A better understanding of the biotic and abiotic control processes of evapotranspiration is necessary for the assessment of local, regional, and global water and carbon budgets as climate change progresses. Studies to quantify the relationship between mass flux and surface conductance at the forest ecosystem level are still needed. Analysis of dry-canopy transpiration measured with the eddy covariance method will provide information on stomatal behaviour and influence on water vapour and CO_2_ fluxes.

Our primary objectives were (1) to examine diurnal and seasonal changes in CO_2_ flux and evapotranspiration, (2) to provide information on the magnitude and temporal variation of parameters representing the bulk canopy characteristics, and (3) to understand the biotic and abiotic control processes of transpiration of Scots pine ecosystem.

## Materials and Methods

### 2.1. Site Description

The research was conducted in a 50-year-old pure stand of Scots pine (*Pinus sylvestris* L.) at Huhus (62°52′N, 30°49′E, 145 m a.s.l.), eastern Finland. The stand density was 1175 trees ha^−1^ (ranging from 7.2 cm to 29.5 cm in diameter at breast height), with a mean height of 11.8 m above the ground and a mean diameter at breast height of 11.2 cm. The leaf area index was about 1.98 when flux measurements commenced. The soil is of a sandy podzol type. The top 50 cm contained an average volumetric mineral fraction of 47% and an organic fraction of 21% and had a mean bulk density of 1.34 g cm^−3^ [19]. The climate is characterized by a long, cold winter. The mean monthly temperature is lowest in January, −10.4°C, and highest in July, 15.8°C. The average annual precipitation at the site (1961–2000) is 724 mm, of which 38% falls as snow. The mean soil moisture (10 cm depth) was lower than 30% that is regarded as roughly representing drought year [30]. The soil moisture (25 cm depth) was lower than 15% in the mid summer (June 21-August 8) of 2003, thus considering as an unusual drought summer. The ground was covered by small patches of litter (30% of the area) or lichen (65% of the area). The understorey is principally mosses (*Dicranum spp*, *Pleurozium schreberi*) and dwarf shrubs (*Vaccinium vitis-idaea*, *Calluna vulgaris*), so the site represents the Calluna type, on a sandy soil with a low nitrogen supply. The field studies did not involve endangered or protected species and no specific permits were required for the described field studies.

The site is flat, and there is a homogeneous underlying surface. The terrain is relatively level, extending at least 2 km around the tower used for the eddy covariance (EC) measurements. It is assumed that zero plane displacement ranges from 7 to 11 m and that the roughness length is between 1 and 2 m. The 80% contribution of the measured flux comes from within 460 m of the upwind area under unstable conditions. This contribution may be from within 1890 m of the upwind area under neutral conditions [20].

### 2.2. Measurements

Half-hour eddy fluxes including CO_2_ flux (Fc), latent heat flux (LE), and sensible heat flux (H), were measured continuously at the top of a 34 m mast, about 20 m above the canopy. Wind velocity and virtual temperature were determined with a Solent 3D ultrasonic anemometer (R2 Gill Instruments, Lymington, UK), and CO_2_ and water vapour concentration fluctuations with a closed-path dual CO_2_/H_2_O analyzer (IRGA; model LI-6262, LiCor, Lincoln, NE, USA). The air was ducted down from a point close to the anemometer to the Li-6262 by means of a sampling tube of length 42 m and radius 3 mm. The air flow rate in the tube was maintained at a constant rate of 6 litres min^−1^ by a mass flow controller (Tylan FC2900B, Tylan General, Swindon, UK) on the sample line. The analogue signals from the Li-6262 were passed to the Solent 3D ultrasonic anemometer, which used an on-board analogue-to-digital converter to digitise the non-linearized signals at 10 Hz. The digitised signals from the Li-6262, combined with the wind speed components (u, v, and w) and the speed of sound, from which air temperature may be derived at 21 Hz, were sent to a computer. The data were collected and processed in real time to provide near-continuous measurements. The EdiSol system was used to calculate the raw data on-line over 30-min interval [21]. Further details of the instrumental installation and calibration are given by Kellomäki and Wang [22].

Simultaneously with the flux measurements, environmental and meteorological variables were measured using a Vaisala weather station (MILOS 500, Vaisala Oy, Helsinki, Finland) as placed at a distance of about 20 m from the eddy covariance mast. Temperature, humidity probes (HMP45D, Vaisala Oy, Helsinki, Finland) and anemometers (WAA15A and WAV15A, Vaisala Oy, Helsinki, Finland) were mounted at 4, 9, 12, and 18 m above the ground along the weather mast to record the weather profile. Canopy temperature was measured with an infrared sensor (IR 4000.4GL, Everest Interscience, Inc. Tucson, USA) and net radiation above the canopy with a combination of an albedometer CM7B (Kipp & Zonen, Delft, Holland) and a CG2 pyrgeometer (Kipp & Zonen, Delft, Holland). Photosynthetically active radiation (PAR) above the canopy was measured with a quantum sensor (LI-190SA), and global radiation at a height of 20 m with a pyranometer (model CM6B/2, Kipp & Zonen, Delft, Holland). Precipitation above and below the forest canopy was measured using 8 rain gauges (RG13, Vaisala Oy, Helsinki, Finland) and bole temperature with copper-constantan thermocouple probes inserted 1 cm into the trunk of each of three trees at heights of 15 cm, 285 cm, and 305 cm above the ground.

Soil heat flux (G) was determined with 4 soil heat flux plates (Radiation Energy Balance System, Seattle, WA, USA) buried 5 cm below the surface in a variety of microenvironments (ranging from mostly sunlit to mostly shaded). The soil volumetric water content (*W_s_*) was monitored at depth of 25 cm below the mineral soil surface with water content reflectometers (CS615, Campbell Scientific, Shepshed, Leics., UK). All of these sensors were sampled at 10 s intervals and the data averaged over 30 min periods using a data logger (21X, Campbell Scientific, Logan, UT, USA).

### 2.3. Post-processing of data

Sonic anemometer measurements were removed when a spiking rate greater than 5 spikes per 30 min interval was observed [23]. These spikes were suspected to occur during periods of heavy rainfall. Flux measurements were also removed when data did not fall within the specified realistic limits and when the non-stationarity ratio was greater than 3.5 [24]. These quality controls resulted in the removal of 1% of the eddy flux measurements. Details of the post-processing of data have been given previously [18]. After data processing, energy balance closure was 70% on the basis of the slope of linear regression of half-hourly heat flux (sum of sensible heat and latent heat flux) against available energy (*R^2^* = 0.68). For post-processed half-hour data, see files of Data S1 and Data S2.

### 2.4. Calculation of bulk parameters

Ecosystem surface conductance to latent heat transfer was calculated by inverting the Penman-Monteith equation [25] as
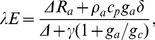
(1)where *g_c_* is the surface conductance (m s^−1^), Δ (kPa K^−1^) the slope of saturated specific humidity versus air temperature (T), *R_a_* the available radiation energy (W m^−2^) as calculated below, *λ* the latent heat of the vaporisation of water (J kg^−1^), *E* the measured evapotranspiration (kg m^−2^ s^−1^), *δ* the vapour pressure deficit (kPa), *ρ_a_* the density of dry air (kg m^−3^), *c_p_* the specific heat of air at constant pressure (J kg^−1^ K^−1^), *λE* the latent heat flux (W m^−2^), and *γ* the psychrometric constant (0.0665 kPa K^−1^). *g_a_* is the aerodynamic conductance (m s^−1^) calculated as [26]

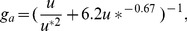
(2)where u is the wind speed above the canopy (m s−1) as measured by the sonic anemometer and u* is the friction velocity (m s−1).

The decoupling coefficient (*Ω*) was subsequently calculated to describe the sensitivity of evapotranspiration to a change in surface conductance [12]. The values of Ω ranges from 0 to 1 with the control of evapotranspiration by surface conductance increasing as Ω approaches 0 [12], [27].

The analyses were conducted for days with a dry canopy (recorded precipitation both above and below the canopy was 0 and PAR more than 200 µmol m^−2^ s^−1^). By excluding data recorded up to 2 days after rain, we minimized inclusion of the evaporation of water that had been intercepted by the canopy [28]. The decoupling coefficient (*Ω*) was calculated according to Jarvis and McNaughton [12],

as 
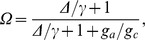
 (3)

The available energy, *R_a_*_,_ was calculated as
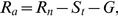
(4)where *R_n_* is the net radiation energy as measured by a Vaisala weather station, *S_t_* the total energy storage in the above-ground air column, and *G* the soil heat flux. The total rate of energy storage (*S_t_*) in a column extending from the ground surface to the EC measurement height *z* was calculated as
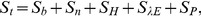
(5)where the subscripts b, n, H, λE and p denote the rates of change in heat content of the stems and leaves, sensible heat content in the air column, latent heat content in the column, and energy consumed in photosynthesis, respectively.

Heat storage in the boles (*S_b_*) was calculated as [29]

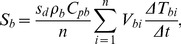
(6)where *s_d_* is the stand density (0.12 stem m^−2^ ground area), *ρ_b_* the average bole density (400 kg m^−3^), Δ*t* the sampling period (30 min) , *V_bi_* the estimated bole volume, and *T_bi_* the bole temperature (K). *V_bi_* was derived from 49 sample trees and *T_bi_* measured from 3 sample trees. *C_pb_* is the average bole specific heat (J kg^−1^ K^−1^), estimated as
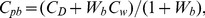
(7)where Wb is the average water content on a dry mass basis (0.968 kg kg−1), CD the specific heat of dry wood (1150 J kg−1 K−1), and Cw the specific heat of water (4190 J kg−1 K−1).

Energy storage in the needles was calculated as
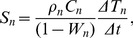
(8)where *W_n_* is the gravimetric water content of the needles on a wet mass basis (55%), *ρ_n_* the average needle density (0.52 kg m^−2^ land area on a dry mass basis [30]), *C_n_* the specific heat of the needles, and Δ*T_n_/*Δ*t* the needle temperature change per half hour. Needle temperature was estimated using the air temperature measured at a height of 12 m within the canopy and assuming that changes in needle temperature would be reflected in the air temperature. Needle specific heat was obtained from 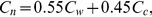
(9)where Cc is the specific heat of cellulose (for glucose 1260 J kg−1 K−1 [31]).

Storage fluxes of sensible heat *H* and latent heat *λE* within the air space beneath the height of turbulent flux measurement were measured from profiles of temperature and relative humidity. The storage terms were then calculated as
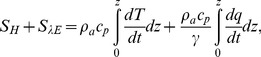
(10)where *γ* is the psychrometric constant (0.0665 kPa K^−1^), *dT/dt* and *dq/dt* are the changes in air temperature (*T*) and water vapour density (*q* ) over the 30-min time period, *ρ_a_* is air density (1.229 kg m^−3^), and *c_p_* is the specific heat of air at constant pressure (1012 J kg^−1^ K^−1^).

The energy consumed in the process of photosynthesis, *S_P_*, was calculated from ecosystem photosynthesis GEP using the photosynthetic energy conversion factor *C* (0.469 J µmol^−1^) [32]) as
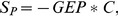
(11)where GEP is the ecosystem photosynthesis in µmol m^−2^ s^−1^ and was calculated by the method described by Zha et al. [33].

Soil heat flux (*G*, W m^−2^) was a sum of measured values (*G_s_*, W m^−2^) by the soil heat plates and heat storage of soil above the soil heat plates as
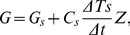
(12)where *Z* (m) is the depth of the layer from plate to surface, *C_s_* the soil's heat capacity estimated by soil water content at *Z*, *T_s_* the soil temperature (average of soil temperature at the depth of 3.5 cm and 2.5 cm below soil surface).

Three typical clear days representing respective spring, summer and autumn were selected to analyze diurnal changes in analyzed variables. Water use efficiency (WUE) was estimated in terms of the reciprocal of the transpiration ratio [34], which is defined as the amount of water transpired by ecosystem divided by the net ecosystem CO_2_ exchange (*F_c_*). For symbols used above, refer to Table 1.

**Table 1:** A list of symbols with their units.

ρ_a_	air density	1.229	kg m^−3^
c_p_	specific heat of air	1012	J kg^−1^ K^−1^
C	photosynthetic energy conversion factor	0.469	J μmol^−1^
C_c_	specific heat of cellulose	1260	J kg^−1^ K^−1^
C_D_	specific heat of dry wood	1150	J kg^−1^ K^−1^
C_n_	specific heat of the needle		J kg^−1^ K^−1^
C_pb_	specific heat of bole		J kg^−1^ K^−1^
C_s_	specific heat of soil		J kg^−1^ K^−1^
C_w_	specific heat of water	4190	J kg^−1^ K^−1^
E	evapotranspiration		kg m^−2^ s^−1^
g_a_	aerodynamic conductance		m s^−1^
g_c_	surface conductance		m s^−1^
G	soil heat flux		W m^−2^
G_s_	measured soil heat flux		W m^−2^
P	ecosystem photosynthesis		µmol m^−2^ s^−1^
q	water vapor density		kg m^−3^
R_a_	available energy		W m^−2^
R_n_	net radiation energy		W m^−2^
s_d_	stand density	0.12	stem m^−2^
S_b_	heat storage in the bole		W m^−2^
S_H_	storage fluxes of sensible heat		W m^−2^
S_n_	heat storage in the needle		W m^−2^
S_P_	energy consumption by photosynthesis		W m^−2^
S_t_	total energy storage		W m^−2^
S_λE_	storage fluxes of latent heat		W m^−2^
t	time		S
T	air temperature		K
T_b_	bole temperature		K
T_n_	needle temperature		K
T_s_	soil temperature		K
u	wind speed		m s^−1^
u*	friction velocity		m s^−1^
V	bole volume		m^3^
W_b_	average water content of the bole	0.968	kg kg^−1^
W_n_	gravimetric water content of the needles	0.55	kg kg^−1^
W_s_	soil water content		m^3^ m^−3^
γ	psychrometric constant	0.0665	kPa K^−1^
Δ	slope of saturated specific humidity versus air temperature		kPa K^−1^
Ω	decoupling coefficient		

### 2.5. Statistical analysis

The significance of the seasonal changes in measured and calculated parameters was analysed by univariate ANOVA, and the degrees to which evapotranspiration, CO_2_ flux, and surface conductance were controlled by environmental variables (PAR, *δ*, *T*, *W_s_*, and *u*) were analysed by stepwise linear regression. The growing season was divided into months for significance test of the seasonal changes. All the statistical analyses were performed on the basis of 24-hour averages using the SPSS 12.0 program for Windows (SPSS Inc., Illinois, USA).

## Results

### 3.1. Seasonal changes in energy components

**Figure 1:**
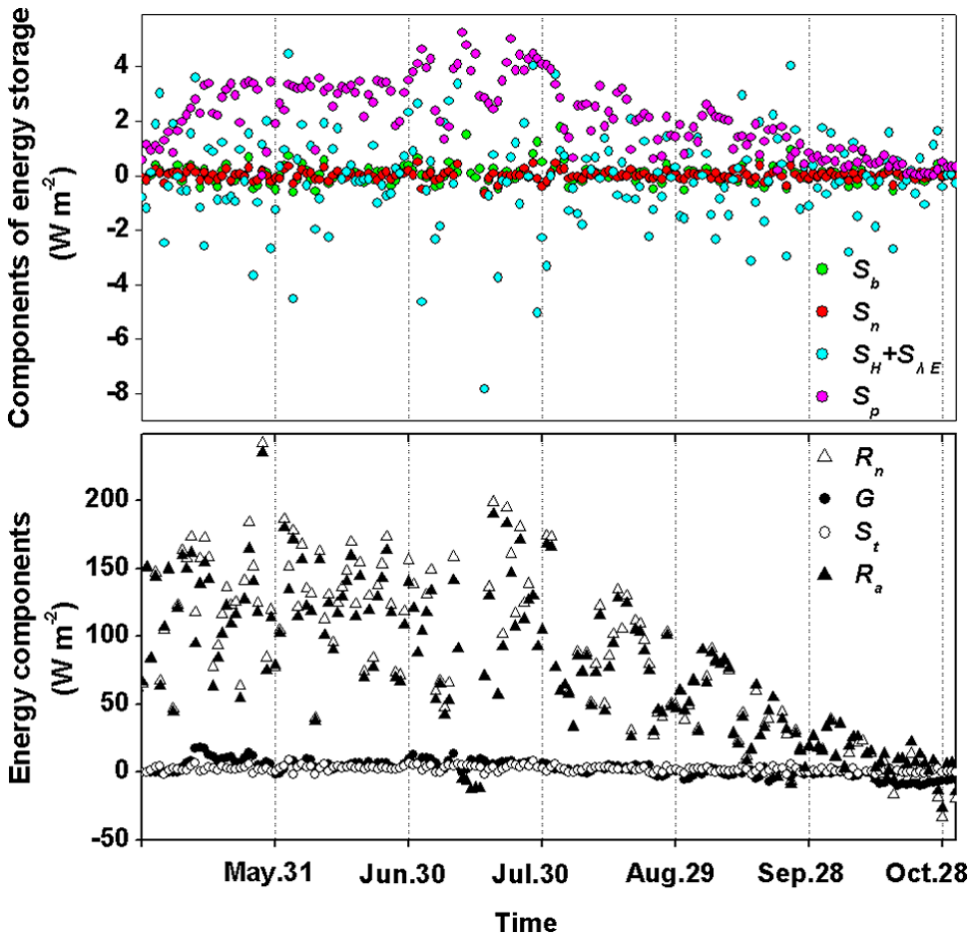
Seasonal changes in net radiation (*R_n_*), soil hear flux (*G*), and total above-ground energy storage (*S_t_*) which include heat content of the stems (*S_b_*), leaves (*S_n_*), sensible and latent heat content in the air column (*S_H_*+*S_λE_*), and energy consumed in photosynthesis from May to October, 2003. Values are 24-h averages.

The upper panel of figure 1 showed the daily mean rates of components of total above-ground energy storage (*S_t_*) over the growing season, including heat content of the stems (*S_b_*), leaves (*S_n_*), sensible and latent heat in the air column (*S_H_+S_λE_*), and energy consumed by photosynthesis (*S_p_*). Among these components, energy use by photosynthesis accounted for the largest portion, being 95% of *S_t_*. There was seasonal change in *S_p_*, *R_n_*, and *R_a_* (Fig. 1). The other energy components fluctuated around zero. The energy closure on average was 70% over the growing season. Addition of soil heat flux only to available energy increased energy closure by 3%, and addition of both soil heat flux and *S_t_* increased by 4%. Therefore energy storage only accounted for little amount of energy over daily time period and can be neglected in energy balance analysis.

### 3.2. Diurnal and seasonal changes

**Figure 2:**
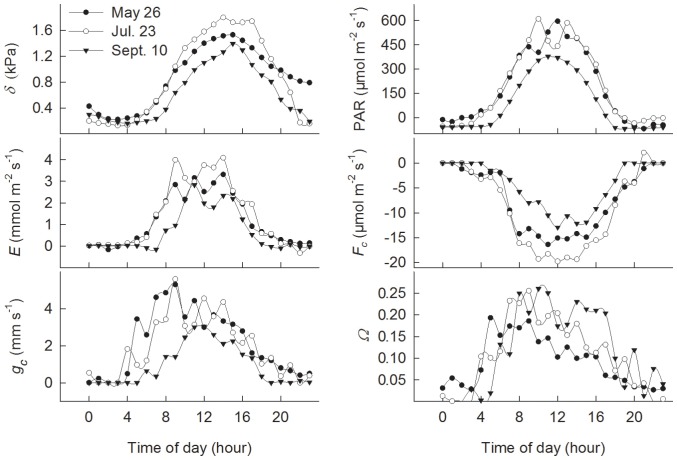
Diurnal changes in evapotransipiration (*E*), CO_2_ flux (*F_c_*), surface conductance (*g_c_*), decoupling coefficient (*Ω*), vapour pressure deficit (*δ*) and radiation (PAR) in a Scots pine forest ecosystem on 3 typical clear days on May 26, July 23 and September 10, 2003.

There were obvious diurnal changes in evapotranspiration (*E*), CO_2_ flux (*F_c_*, minus represents CO_2_ into the canopy), ecosystem surface conductance (*g_c_*), and decoupling coefficient (*Ω*), showing a diurnal and seasonal pattern that was marginally more similar to those of PAR than to vapour pressure deficit (*δ*) (Fig. 2). The values of all the variables were low at night (closer to 0 before 3 h and after 18 h) and high during the mid-day period from 8 to 16 h. The magnitudes of *E*, *F_c_*, and *g_c_* were lower on September 10 than those on the other two days as a whole, showing a correspondence with PAR. *Ω* values were lower on May 26 than on the other two days.

**Figure 3:**
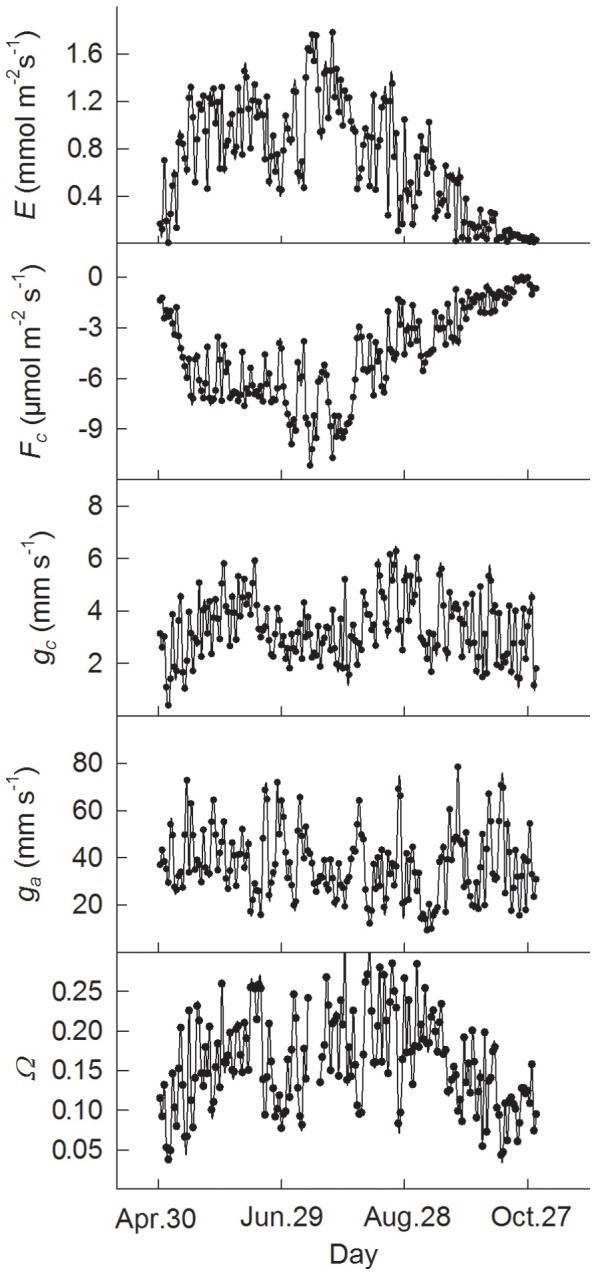
Seasonal changes in evapotranspiration (*E*), ecosystem CO_2_ Flux (*F_c_*), surface conductance (*g_c_*), aerodynamic conductance (*g_a_*), and decoupling coefficient (*Ω*) in a Scots pine forest ecosystem from May to October, 2003. Values are 24-h averages.

**Figure 4:**
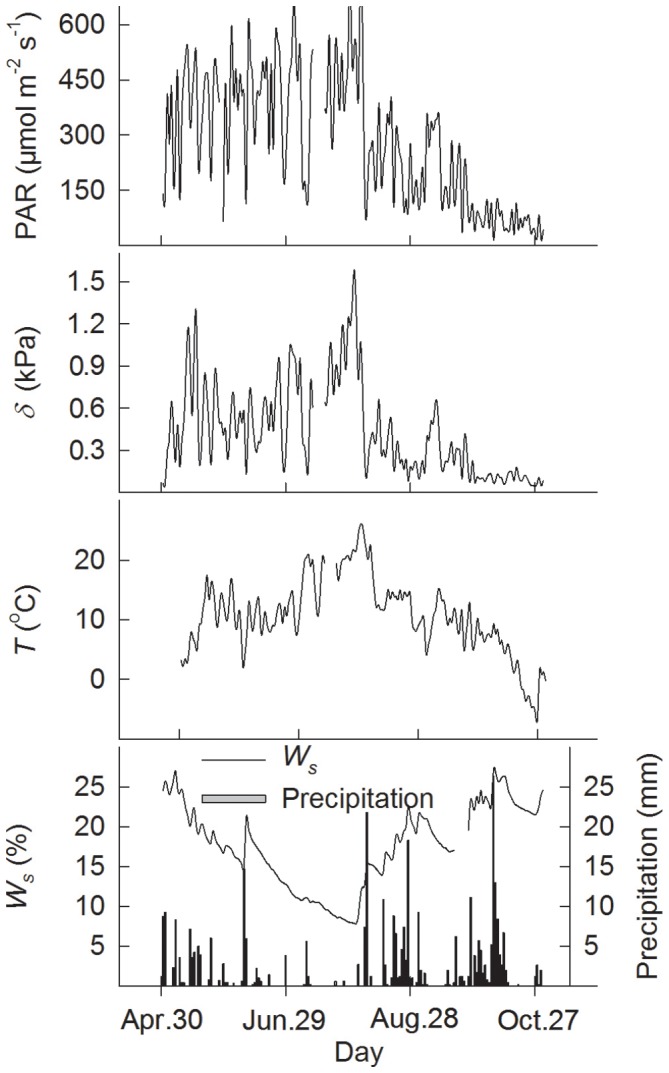
Mean daily values for radiation (PAR), vapour pressure deficit (*δ*), air temperature at 18.4 m above the ground (*T*) and water content of the upper 25 cm of the soil (*W_s_*), and daily total precipitation from May to October, 2003.

Mean daily values (24-h average) from May to October indicated significant seasonal changes in *E*, *F_c_*, *g_c_*, and *Ω* (Fig. 3, P<0.0001), with seasonal trends similar to those in the environmental variables PAR, *δ,* and air temperature (*T*) (Fig. 4). Overall, these physiological variables had high values from June to September, with the exception of low values during drought period from late June to July, when there was little rainfall and the soil water content (*W_s_*) was low (Fig. 4). The *g_c_* remained relatively constant during drought period from late June to July (Fig. 3). The mean daily values for *E*, *F_c_*, *g_c_*, and *Ω* ranged from 0.0038 to 1.78 mmol m^−2^ s^−1^, −0.0061 to −11.18 µmol m^−2^ s^−1^, 0.4 to 6.27 mm s^−1^, and 0.03 to 0.31, respectively, and averaged 0.71±0.04, −4.61±0.12, 3.3±0.09, and 0.16±0.004. The highest values for *E* and *F_c_* occurred in July, when LAI was highest [16]. All the variables had low values in May and October. Aerodynamic conductance (*g_a_*) remained relatively constant, without any significant seasonal changes, despite short-term fluctuations (Fig. 3, *P* = 0.088).

### 3.3. Control of evapotranspiration

**Figure 5:**
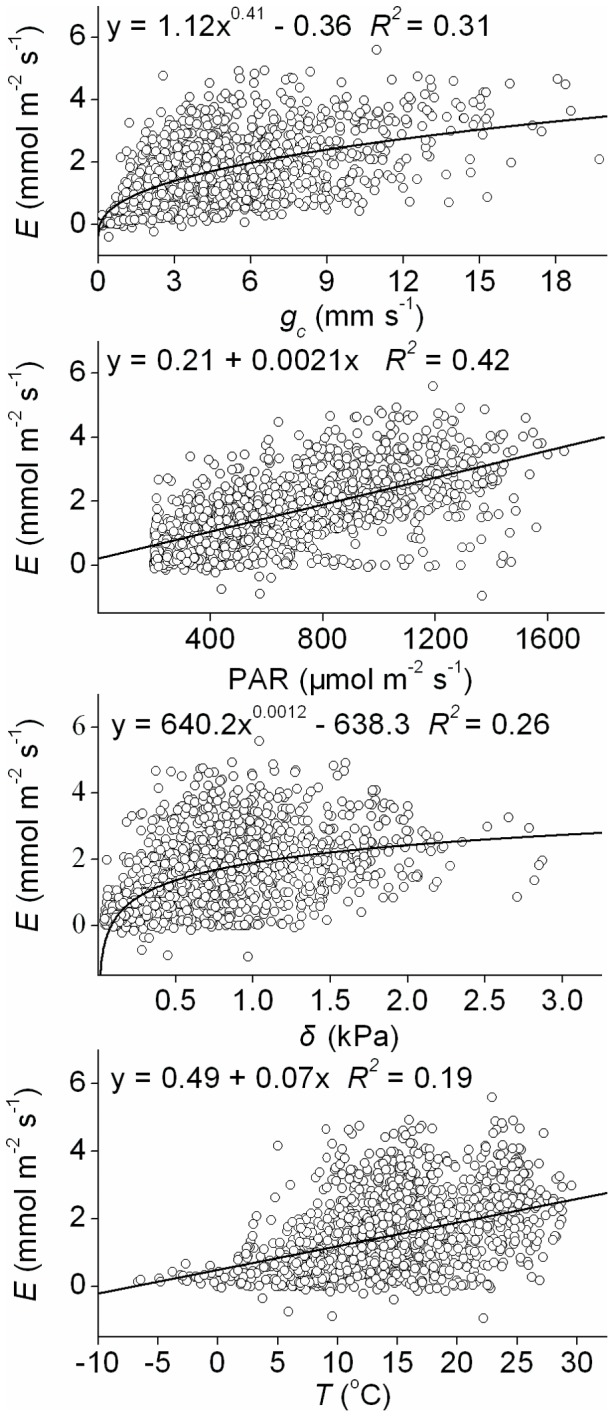
Evapotranspiration (*E*) as a function of surface conductance (*g_c_*), radiation (PAR), vapour pressure deficit (*δ*), and air temperature (*T*) from May to October, 2003. Values are hourly averages on a dry surface (PAR >200 µmol m^−2^ s^−1^, with zero precipitation above and under the canopy).

The relationship between ecosystem evapotranspiration (*E*) and biological variables, represented by surface conductance (*g_c_*) during periods when the leaves were dry (PAR >200 µmol m^−2^ s^−1^ and total hourly rainfall both above the canopy and under the canopy were 0), and corresponding environmental variables are shown in Fig. 5. *E* increased curvilinearly with increasing *g_c_* and vapour pressure deficit (*δ*) (Fig. 5), with *g_c_* and *δ* explaining 33% and 26% of the variation in *E*, respectively. It was also linearly related to radiation (PAR) and air temperature (*T*). PAR, *δ*, and *T* individually explain 42%, 26%, and 19% of the variation in *E*, respectively. No meaningful relationship between *E* and soil water content (*W_s_*) or wind speed (*u*) could be found.

Stepwise linear regression of evapotranspiration against the environmental variables (PAR, *δ*, *T*, *W_s_*, and *u*) shows that *E* was controlled by PAR, *T*, *δ*, and *u* together (*R^2^* = 0.48, n = 1410). Net radiation had more effect on *E* than did the other environmental variables.

When the biological variable *g_c_* and environmental factors were considered together as independent variables in the stepwise linear regression while *E* was taken as a dependent variable, 80% of the variation in *E* was explained by PAR, *g_c_*, *δ*, and *T* together (*R^2^* = 0.80, *P*<0.0005).

**Figure 6:**
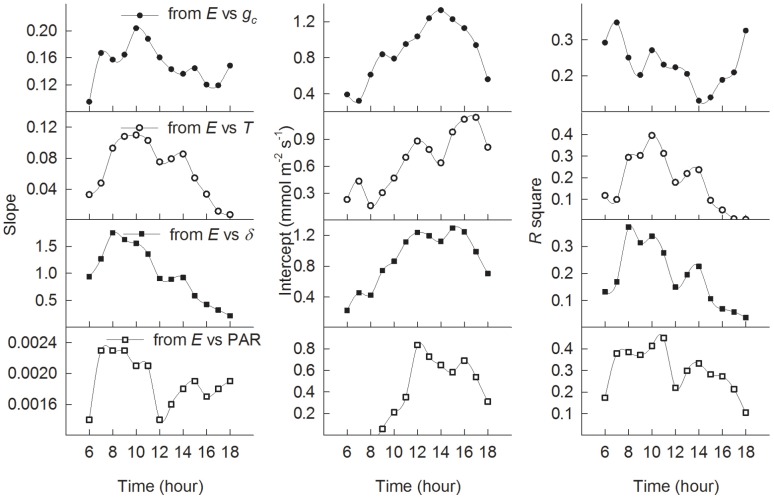
Parameters for the linear regressions between evapotranspiration (*E*) and surface conductance (*g_c_*), air temperature (*T*), vapour pressure deficit (*δ*), and radiation (PAR) at given times from 6 to 18 h throughout the growing season. The regression values are calculated on the basis of a dry surface (PAR >200 µmol m^−2^ s^−1^, with zero precipitation above and under the canopy).

In order to examine the daily variations in the sensitivity of *E* to the variables (PAR, *g_c_*, *δ*, and *T*) and variations in their relationship, we plot the slopes, intercepts and *R*-square of their regression lines over the growing season. As a result, the values of the slopes for the linear regression between *E* and *g_c_*, *T*, *δ*, and PAR were higher in the morning, from 8 to 11 h, than in the afternoon, after 12 h (Fig. 6). Similarly, *R*-square exhibited higher values in the morning than in the afternoon, from 8 to 11 h. The high values for the intercept occurred after 11 h.

### 3.4. Control of carbon flux

**Figure 7:**
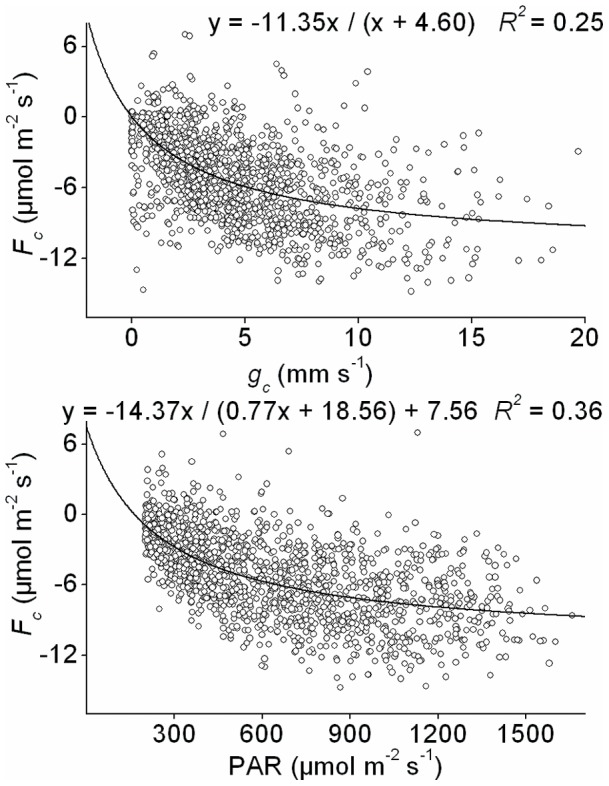
Ecosystem CO_2_ flux (*F_c_*) as a function of surface conductance (*g_c_*) and PAR. Values are hourly averages from May to October, 2003, calculated on the basis of a dry surface (PAR >200 µmol m^−2^ s^−1^, with zero precipitation above and under the canopy).

*F_c_* (minus represents CO_2_ flux into the canopy) increased with increasing surface conductance (*g_c_*) (Fig. 7), showing a curvilinear relationship (*R^2^* = 0.28). Among the environmental factors (PAR, *δ*, *T*, *W_s_*, and *u*), PAR was the most important factor controlling *F_c_*, which increased curvilinearly with increasing PAR (*R^2^* = 0.45). Stepwise linear regression of *F_c_* against the environmental variables showed that PAR, *δ*, *u*, and *W_s_* together explain 43% of the variation in *F_c_* (*R^2^* = 0.41, n = 1413). When the biological variable *g_c_* and environmental variables are taken together as independent variables in stepwise linear regression, 54% of the variation in *F_c_* is explained by PAR, *g_c_*, *δ*, *u*, and *W_s_* together. Temperature had the weakest correlation with *F_c_*.

### 3.5. Control of surface conductance

**Figure 8:**
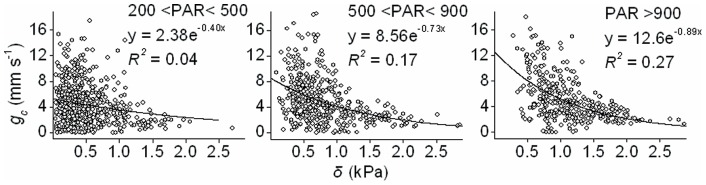
Surface conductance (*g_c_*) as a function of vapour pressure deficit (*δ*) in three PAR (µmol m^−2^ s^−1^) classes. Values are hourly averages from May to October calculated on the basis of a dry surface (PAR >200 µmol m^−2^ s^−1^, with zero precipitation above and under the canopy).

**Figure 9:**
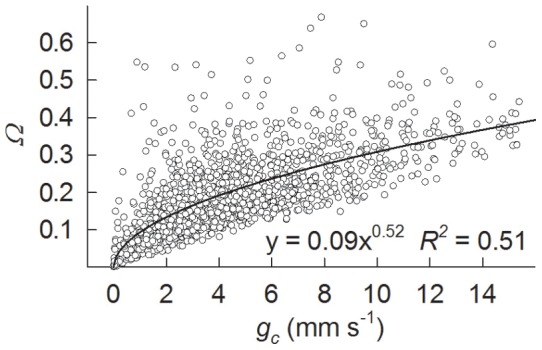
Decoupling coefficient (*Ω*) as a function of surface conductance (*g_c_*). Values are hourly averages from May to October, 2003, calculated on the basis of a dry surface (PAR >200 µmol m^−2^ s^−1^, with zero precipitation above and under the canopy).

Surface conductance (*g_c_*) is controlled by vapour pressure deficit (*δ*), radiation (PAR), air temperature (*T*), and wind speed together (stepwise linear regression, *R^2^* = 0.17, n = 809). Among the environmental factors, *δ* had the closest correlation with *g_c_*, a better fit being achieved with the exponential equation (Fig. 8, *R^2^* = 0.27). The *g_c_* decreased with increasing *δ* in the regression analysis. The slopes of the regression curves are steep at high PAR, i.e. 0.89 at PAR >900 µmol m^−2^ s^−1^ and 0.73 at PAR between 500–900 µmol m^−2^ s^−1^. Surface conductance also affected the decoupling coefficient *Ω*, which increased in a non-linear manner with increasing *g_c_* (Fig. 9; *P*<0.0001).

### 3.6. Water use efficiency

**Figure 10:**
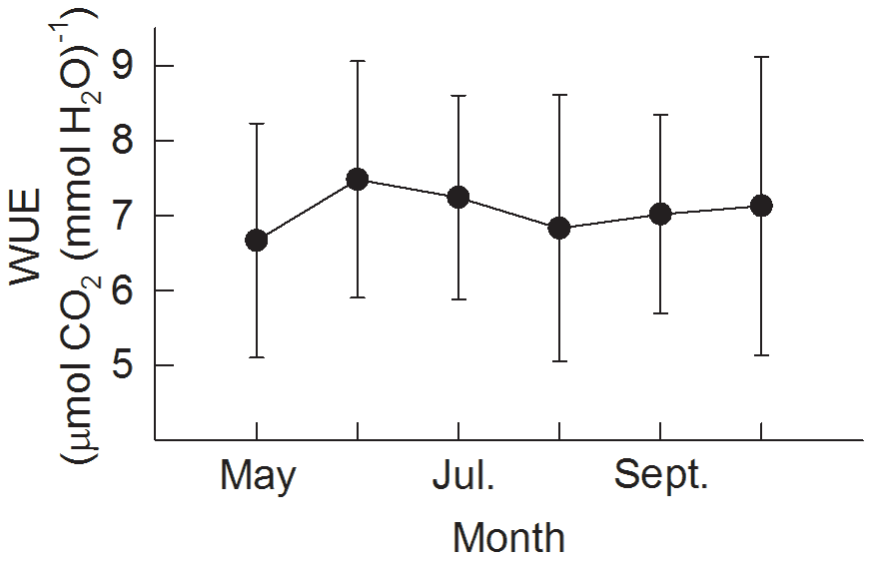
Water use efficiency (WUE) from May to September, calculated on the basis of a dry surface (PAR >200 µmol m^−2^ s^−1^, with zero precipitation above and under the canopy). Values are monthly averages. The error bars represent standard deviation.

WUE was calculated on the days when leaves were dry (PAR >200 µmol m^−2^ s^−1^ and total hourly rainfall both above canopy and under the canopy were 0). The mean monthly values of WUE ranged from 6.67 μmol CO_2_ (mmol H_2_O)^−1^ in May to 7.48 in October, with a mean value of 7.06 (Fig. 10).

## Discussion

### 4.1. Diurnal and seasonal changes of physiological parameters

The trend in diurnal and seasonal changes in the physiological parameters (evapotranspiration *E*, ecosystem CO_2_ flux *F_c_*, surface conductance *g_c_*, and decoupling coefficient *Ω*) was consistent with that in radiation. It was noted that the little rainfall and low soil water content from late June to early July led to lower values of *E*, *g_c_*, and *Ω* (Fig. 3, 4). One possible explanation would be that the stomatal pores closed under conditions of a soil water deficiency in order to prevent excessive water loss, thereby leading to a reduction in transpiration. Lower and relatively constant *g_c_* further support that stomata was closed under severe drought condition from late June to July. This supports the conclusion by Duursma [35] that a model with constant plant conductance and minimum leaf water potential can accurately predict the decline in daily maximum transpiration rate during drought. *F_c_* remained high in the main growing season from June to August. This could be explained by high LAI [18]. Another explanation might be that stomata closed to some degree in response to greatly vapour pressure deficit (*δ*), but photosynthesis proceeded at a slightly higher WUE and without any metabolic limitation.

### 4.2. Control of evapotranspiration

The present analysis of environmental control over evapotranspiration in a Scots pine ecosystem showed that radiation, water vapour pressure deficit and temperature are the main environmental factors involved (*R^2^* = 0.48), with radiation the most important. However, since radiation alone explained 42% of the variation in evapotranspiration, it is suggested that ecosystem evapotranspiration cannot be adequately predicted by this alone, despite its importance.

Previous studies have shown that evapotranspiration is controlled by interaction between a number of environmental and biological factors [15], [16], [36], [37]. Biological control can generally be represented by surface conductance [8], [10], [15], which is thus taken here as a representative of biological variables. The basic explanation for this is the following. The low values for the decoupling coefficient (*Ω*) (Fig. 3) imply that the canopy was well coupled to the atmosphere, so that it can be assumed that the calculation of surface conductance does not violate the assumption of the Penmen-Monteith equation in which canopy is treated as a single layer [38]. Although surface conductance calculated from the Penmen-Monteith equation does not explicitly represent the biological parameter known as stomatal conductance, because theoretical and experimental investigations indicate that surface conductance is often related to the weighted integration from individual leaves [39], [40]. Surface conductance can therefore reflect biological control of ecosystem transpiration.

The finding that surface conductance and environmental variables including radiation, vapour pressure deficit and air temperature together explained 80% of the variation of evapotranspiration, which is much more than that achieved by the environmental factors either together (48%) or separately (19%–42%), suggests that ecosystem evapotranspiration is codetermined both biologically and environmentally. Surface conductance alone explained 33% of the variation of evapotranspiration in the present case. Results in the literature suggest that transpiration flux from coniferous forests in the boreal region is largely controlled by canopy conductance [12]. This is because boreal forest canopies are aerodynamically rough and well ventilated, which minimizes the effect of aerodynamic conductance. The relatively constant figures obtained here for aerodynamic conductance (g_a_, Fig. 3) provide further support for this.

The decoupling coefficient *Ω* has also been widely used to determine the relative importance of surface conductance and net radiation for changes in evapotranspiration [41]. The low values of *Ω* recorded here, which are similar to those in temperate coniferous forests [23], indicate that evapotranspiration is highly sensitive to surface conductance. On a daily basis, *Ω* was highest in the morning and lowest in the evening (Fig. 1), indicating tighter control over water loss by plants as the day progressed. The close relationship between *Ω* and *g_c_* further supported the importance of biological control with regard to changes in evapotranspiration (Fig. 9; *R^2^* = 0.51, *P*<0.0001).

High morning values for the slope and R-square of the linear regression of evapotranspiration against surface conductance and environmental variables at a given time indicate that evapotranspiration is more sensitive to biological and environmental factors in the morning than in the afternoon (Fig. 6). The explanation could be that the wide open stomatal pores lead to a greater water flux into the atmosphere in the morning, and thus to greater transpiration or greater biological control over evapotranspiration than in the mid-day period, when the stomata are less open.

### 4.3. Control of CO_2_ flux

In our work, surface conductance and environmental variables (radiation, vapour pressure deficit, wind speed, and soil water content) together explained 54% of the variation in CO_2_ flux *F_c_* in a linear manner as compared with 41% in the case of the four environmental variables together and 45% for PAR alone, indicating a dominant control of canopy conductance over *F_c_*. The main reason for this is that both water vapour and CO_2_ pass through the stomata. Hence, the stomata of the canopy are not only the key to the assessment of transpiration and water balance, but are also important for the estimation of carbon flux.

We did not find any meaningful relationship between *F_c_* and any other environmental factors except for PAR, which was linearly related to CO_2_ flux (Fig. 7). A close relationship between PAR and *F_c_* has been found previously [33]. The low value for R-square in the regression between PAR and CO_2_ flux is because that the regression applies only to the daytime, when PAR is more than 200 µmol m^−2^ s^−1^ from May to October. We thought that the control of CO_2_ flux by radiation would decrease when radiation remained higher than 200 µmol m^−2^ s^−1^ and was no longer a limited factor for photosynthesis in the Scots pine ecosystem.

### 4.4. Control of surface conductance

The finding that surface conductance decreased with increasing vapour pressure deficit agrees with previous reports [14], [42] in which the surface and canopy conductance decline exponentially with increasing vapour pressure deficit in a variety of leaf, whole-tree and stand-level studies. The values for the slopes in Fig. 8 imply that the sensitivity of surface conductance to changes in vapour pressure deficit is high under conditions of more pronounced radiation.

Vapour pressure deficit was the most important environmental factor affecting the surface conductance, explaining much more of its variation than temperature, radiation, or soil water potential, as also reported by Gunderson *et al.* [43]. Although the mechanism of the stomatal response to vapour pressure deficit is unknown, some studies suggest that this response occurs as a feedback related to transpiration and water loss from the leaf rather than as a direct response to humidity [41], [45]. We noted that there was no relationship between surface conductance and any other environmental factors except for vapour pressure deficit, the possible reason being that surface conductance is mostly regulated by the plant itself when environmental conditions are favourable. Whatever the explanation is, plants regulate their conductance in order to optimize photosynthesis while minimizing water loss through their leaves [44], [13], [46]. WUE was high in June and July when rainfall and soil water content were low (Fig. 10), indicating the role of the stomata in minimizing water loss and maximizing CO_2_ flux in dry season.

## Conclusions

Obvious seasonal changes in evapotranspiration and CO_2_ flux were partially driven by radiation, vapour pressure deficit, and temperature. Radiation had more influence on both evapotranspiration and CO_2_ flux than did the other environmental factors. Soil water deficiency in mid summer led to a lower and constant surface conductance during drought and a lower decoupling coefficient, thereby reducing transpiration. Vapour pressure deficit was the most important factor affecting surface conductance which was more sensitive to vapour pressure deficit under conditions of high radiation. Higher WUE in dry condition indicated an acclimation of plant to water deficiency.

## Supporting Information

DataHuhus2003 S1 Half-hour fluxes and meteorological data for Scots pine ecosystem at Huhus in Finland in 2003.
(XLS)

MetadataHuhus2003 S2 Documentation of the file of halfhour data for Scots pine ecosystem at Huhus in Finland in 2003.
(DOC)

## References

[1] Carter TR, Bärlund I, Fronzer S, Kankaanpää S, Kaivio-oja J, et al.. (2002) The finnish global change scenarios. In: Käyhkö J, Talve L (Ed.) Understanding the Global System. The Finnish Perspective. Painosalama, Turku.

[2] Hall FG (1999) Introduction to special section: BOREAS in 1999: experiment and science overview. J Geophys Res 104: 27627–27639.

[3] Hari P, Mäkelä A (2003) Annual pattern of photosynthesis in Scots pine in the boreal zone. Tree physiology 23: 145–155. 12566265 10.1093/treephys/23.3.145

[4] Scott RL, Huxman TE, Cable WL, Emmerich WE (2006) Partitioning of evapotranspiration and its relation to carbon dioxide exchange in a Chihuahuan Desert shrubland. Hydrological Processes 20 (15): 3227–3243.

[5] Stoy PC, Katul GG, Siqueira MBS, Juang J-Y, Novick KA, et al (2006) Separating the effects of climate and vegetation on evapotranspiration along a successional chronosequence in the southeastern US. Global Chang Biol 12: 2115–2135.

[6] Sauer TJ, Singer JW, Prueger JH, DeSutter TM, Hatfield JL (2007) Radiation balance and evaporation partitioning in a narrow-row soybean canopy. Agric For Meteorol 145 (3–4): 206–214.

[7] Hu ZM, Yu GR, Zhou YL, Sun XM, Li YN, et al (2009) Partitioning of evapotranspiration and its controls in four grassland ecosystems: Application of a two-source model. Agric For Meteorol 149(9): 1410–1420.

[8] Zha TS, Barr AG, Kamp GVD, Black TA, McCaughey JH, et al (2010) Interannual variation of evapotranspiration from forest and grassland ecosystems in western Canada in relation to drought. Agric For Meterol 150: 1476–1484.

[9] Zhang YQ, Kang SZ, Ward EJ, Ding RS, Zhang X, et al (2011) Evapotranspiration components determined by sap flow and microlysimetry techniques of a vineyard in northwest China: Dynamics and influential factors. Agr Water Manage 98: 1207–1214.

[10] Kumagai T, Saitoh TM, Sato Y, Morooka T, Manfroi OJ, et al (2004) Transpiration, canopy conductance and the decoupling coefficient of a lowland mixed dipterocarp forest in Sarawak, Borneo: dry spell effects. J Hydrol 287: 237–251.

[11] Yoshida M, Ohta T, Kotani A, Maximov T (2010) Environmental factors controlling forest evapotranspiration and surface conductance on a multi-temporal scale in growing seasons of a Siberian larch forest. J Hydrol 395 (3–4): 180–189.

[12] Jarvis PG, McNaughton KG (1986) Stomatal control of transpiration: scaling up from leaf to region. Adv Ecol Res 15: 1–49.

[13] Berninger F, Mäkelä A, Hari P (1996) Optimal control of gas exchange during drought: empirical evidence. Ann Bot 77: 469–476.

[14] Wullschleger SD, Gunderson CA, Hanson PJ, Wilson KB, Norby RJ (2002) Sensitivity of stomatal and canopy conductance to elevated CO2 concentration – interacting variables and perspectives of scale. New Phytol 153: 485–496. 10.1046/j.0028-646X.2001.00333.x 33863220

[15] Wilson KB, Baldocchi DD (2000) Seasonal and interannual variability of energy fluxes over a broadleaved temperate deciduous forest in North America. Agric For Meterol 100: 1–18.

[16] Wu SH, Jansson P-E, Kolari P (2011) Modeling seasonal course of carbon fluxes and evapotranspiration in response to low temperature and moisture in a boreal Scots pine ecosystem. Ecol Model 222(17): 3103–3119.

[17] Falge E, Baldocchi D, Tenhunen J, Aubinet M, Peter B, et al (2002) Seasonality of ecosystem respiration and gross primary production as derived from FlUXNET measurements. Agric For Meteorol 113: 53–74.

[18] Wang K-Y, Kellomäki S, Zha TS, Peltola H (2004) Seasonal variation in energy and water fluxes in a pine forest: an analysis based on eddy covariance and an integrated model. Ecol Model 179: 259–279. 10.1093/treephys/24.1.19 14652211

[19] Kellomäki S, Wang KY (1999) Short-term environmental controls of heat and water vapour fluxes above a boreal coniferous forest: model computations compared with measurements by eddy correlation. Ecol Model 124: 145–173.

[20] Wang QG (2003) Measurements and modeling the evapotranspiration of a Scots pine forest. PhD thesis. University of Joensuu, Finland.

[21] Moncrieff JB, Massheder JM, De Bruin H, Elbers J, Friborg T, et al (1997) A system to measure surface fluxes of momentum, sensible hear. J Hydrol 188/189: 589–611.

[22] Kellomäki S, Wang KY (2000) Short-term environmental controls on carbon dioxide flux in a boreal coniferous forest: model computation compared with measurements by eddy covariance. Ecol. Model. 128: 63–88.

[23] Humphreys ER, Black TA, Ethier GJ, Drewitt GB, Spittlehouse DL, et al (2003) Annual and seasonal variability of sensible and latent heat fluxes above a coastal Douglas-fir forest. British Columbia, Canada. Agric For Meteorol 115: 109–125.

[24] Mahrt L (1998) Flux sampling errors for aircraft and towers. J Ocean Atmos Tech 15: 416–429.

[25] Stewart JB (1988) Modelling surface conductance of pine forest. Agric For Meteorol 43: 19–15.

[26] Monteith JL, Unsworth MH (1990) Principles of Environmental Physics. 2nd Edition. Chapman and Hall, New York, USA.

[27] Meinzer FC (1993) Stomatal control of transpiration. Trends in Ecology and Evolution 8, 289–294. 10.1016/0169-5347(93)90257-P 21236171

[28] Wever LA, Flanagan LB, Carlson PJ (2002) Seasonal and interannual variation in evapotranspiration, energy balance and surface conductance in a northern temperate grassland. Agri For Meteorol 112: 31–49.

[29] Turnipseed AA, Blanken PD, Anderson DE, Monson RK (2002) Energy budget above a high-elevation subalpine forest in complex topography. Agric For Meteorol 110: 177–201.

[30] Helmisaari H-S, Makkonen K, Kellomäki S, Valtonen E, Mälkönen E (2002) Below- and above-ground biomass, production and nitrogen use in Scots pine stands in eastern Finland. For Ecol Manag 165: 317–326.

[31] Ganster J, Fink H-P (1999) Physical constants of cellulose. In: Brandup J, Immergut EH, Grulke EA (Eds.), Polymer Handbook, 4th Edition. Wiley, New York.

[32] Blanken PD, Black TA, Yang PC, Neumann HH, Nesic Z, et al.. (1997) Energy balance and canopy conductance of a boreal aspen forest: partitioning overstory and understory components. J Geophys Res 102 (D24) 28915–28917.

[33] Zha TS, Kellomäki S, Wang K-Y, Rouvinen I (2004) Carbon sequestration and ecosystem respiration for 4 years in a Scots pine forest. Global Change Biol 10: 1492–1503.

[34] Taiz L, Zeiger E (1999) Plant Physiology. The Benjamin/Cummings Publishing Company. Inc. Redwood city, California.

[35] Duursma RA, Kolari P, Perämäki M, Nikinmaa E, Hari P, et al (2008) Predicting the decline in daily maximum transpiration rate of two pine stands during drought based on constant minimum leaf water potential and plant hydraulic conductance. Tree Physiol 28: 265–276. 18055437 10.1093/treephys/28.2.265

[36] Oren R, Ewers BE, Todd P, Phillips N, Katul G (1998) Water balance delineates the soil layer in which moisture affects canopy conductance. Ecol Application 8: 990–1002.

[37] Brümmer C, Black A, Jassal RS, Grant NJ, Spittlehouse DL, et al (2012) How climate and vegetation type influence evapotranspiration and water use efficiency in Canadian forest, peatland and grassland ecosystems. Agric For Meteorol 153: 14–30.

[38] Raupach MR, Finnigan JJ (1998) Single-layer models of evaporation from plant canopies are incorrect but useful, whereas multilayer models are correct but useless: discuss. Aust J Plant Physiol 15: 705–716.

[39] Paw UKT, Meyers TP (1989) Investigations with a higher order canopy turbulence model into mean source-sink levels and bulk canopy resistance. Agric For Meteorol 47: 259–271.

[40] Baldocchi DD, Meyers TP (1998) On using eco-physiological, micrometeorological and biogeochemical theory to evaluate carbon dioxide, water vapor and trace gas fluxes over vegetation: a perspective. Agric For Meteorol 90: 1–25.

[41] Meinzer FC, Hinckley TM, Ceulemans R (1997) Apparent responses of stomata to transpiration and humidity in a hybrid poplar canopy. Plant Cell Environ 20: 1301–1308.

[42] Addington RN, Mitchell RJ, Oren R, Donovan LA (2004) Stomatal sensitivity to vapor pressure deficit and its relationship to hydraulic conductance in Pinus palustris. Tree Physiol 24: 561–569. 14996660 10.1093/treephys/24.5.561

[43] Gunderson CA, Sholtis JD, Wullschleger SD, Tissue DT, Hanson PJ, et al (2002) Environmental and stomatal control of photosynthetic enhancement in the canopy of sweetgum (Liquidambar styraciflua L.) plantation during three years of CO2 enrichment. Plant Cell Environ 25: 379–394.

[44] Ponton S, Flanagan LB, Alstad KP, Johnson BG, Morgenstern K, et al (2006) Comparison of ecosystem water-use efficiency among Douglas-fir forest, aspen forest and grassland using eddy covariance and carbon isotope techniques. Global Change Biol 12: 294–310.

[45] Buckley TN, Mott KA, Farquhar GD (2003) A hydromechanical and biochemical model of stomatal conductance. Plant Cell Environ 26: 1767–1785.

[46] Katul G, Manzoni S, Palmroth S, Oren R (2010) A stomatal optimization theory to describe the effects of atmospheric CO2 on leaf photosynthesis and transpiration. Ann Bot 105: 431–442. 19995810 10.1093/aob/mcp292 PMC2826246

