# Supplementary material for: Controls of Evapotranspiration and CO2 Fluxes from Scots Pine by Surface Conductance and Abiotic Factors
Source: PLoS One. 2013 Jul 24;8(7):e69027. doi: 10.1371/journal.pone.0069027 (PMC3722186; doi:10.1371/journal.pone.0069027)
Supplement: MetadataHuhus2003 S2 — Documentation of the file of halfhour data for Scots pine ecosystem at Huhus in Finland in 2003. (DOC) [file pone.0069027.s002.doc]

**1. Half-hour data documentation for meteorological factors (sheet ‘CO2&H2O flux’ in the file ‘DataHuhus2003 S1’) at Huhus site, in Finland in 2003**

# Principal Investigator/ Institutional Affiliation

Dr. Seppo Kellomäki

Faculty of Science and Forestry,

University of Eastern Finland, FIN-80101 Joensuu, Finland

e-mail: [seppo.kellomaki@uef.fi](mailto:seppo.kellomaki@uef.fi) Telephone: 0405032145

# Site Location and Description

The research was conducted in a 50-year-old stand of Scots pine (*Pinus* *syvestris* L.) at Huhus (62º52'N, 30º49'E, 145 m a.s.l.), eastern Finland. The stand density was 1176 trees ha^-1^ (ranging from 7.2 cm to 29.5 cm in diameter at breast height), with a mean height of 13.5 m above the ground and a mean diameter at breast height of 11.2 cm. The leaf area index was about 1.98 m^2^ m^-2^ ground area when flux measurements commenced. The soil is of a sandy podzol type. The top 50 cm contained an average volumetric mineral fraction of 47% and organic matter fraction of 21% and had a mean bulk density of 1.34 g cm^-3^. The climate is characterized by a long, cold winter. The mean monthly temperature is lowest in January, -10.4ºC, and highest in July, 15.8ºC. The average annual precipitation at the site (1961-2000) is 724 mm, of which 38% falls as snow. The understorey is principally mosses (*Dicranum spp, pleurozium schreberi*) and dwarf shrubs (*Vaccinium vitis-idaea, Calluna vulgaris*), so that the site represents the *Calluna* type, on a sandy soil with a low nitrogen supply.

The site is flat and there is a homogeneous underlying surface. The terrain is relatively level, extending at least 2 km around the tower used for the eddy covariance (EC) measurements. It is assumed that zero plane displacement ranges from 6.75 to 10.53 m and the roughness length is between 1.01 and 2.16 m. The fetch is about 460 m under unstable conditions and about 1890 m under neutral conditions.

# Methods for water and CO2 exchange

| - **Licor LI-6262** carbon dioxide and water analyzer  - CO2 measuring range 0...1000 ppm  - CO2 accuracy ± 2 ppm - H2O measuring range 0...7.5 kPa - H2O accuracy 1 % from reading - **FC- 2900**  mass flow controller - **Pumps** - **Acernote 350E** portable computer  - Pentium 100 Mhz/8Mb - Hard disk 400 Mb - PCMCIA interface card - Battery for electricity brakes - Chemicals needed by carbon dioxide and water analyzer - Multimeter for checking | 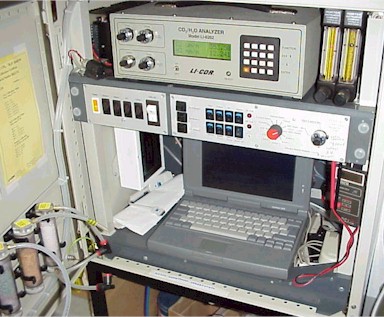 |
| --- | --- |

| Air flowing in the system     - Reference air is dryed by magnesium perchlorate hydrate and carbon dioxide is eliminated by soda lime. In this way we get dry and non carbon dioxide air for comparing. - Sample gas is taken from the top of the 34 m high mast. - First it is dust and trash filtered. - The analyzer is measuring carbon dioxide and water content. - Because pressure of sample gas has influence to analyzer readings, so pressure is measured and analyzer can calculate right values. - Mass flow controller keeps sample gas flowing steady (6 l/minute) - Pump locates after analyzer. - Measuring time constant is 450 s. | 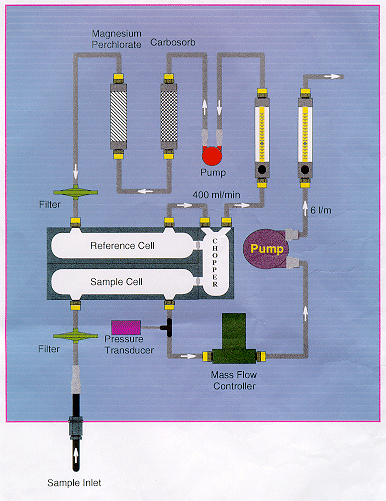 | |
| --- | --- | --- |
| **Solent 1012R2A ultrasonic anemometer** is measuring wind direction and speeds vertically and horizontally. There are three ultrasonic pairs of transmitter and receiver. Because velocity of sound changes at same time as wind speed, so we can calculate wind speed by using the elapsed time between sensors. We get XYZ wind speeds. We can also calculate air temperature by using those speeds.  Ultrasonic anemometer is located on the top of the higher mast a (34,6 m).     - Measuring range 0...60 m/s - Accuracy ± 1,5 % - Max. sample frequency 168 samples/s/vektor - Max. RS-232 transmit frequency 21 measurements/s - Max. with analogical connection 10 samples/s - Measurement temperature range -20°C...+50°C | | 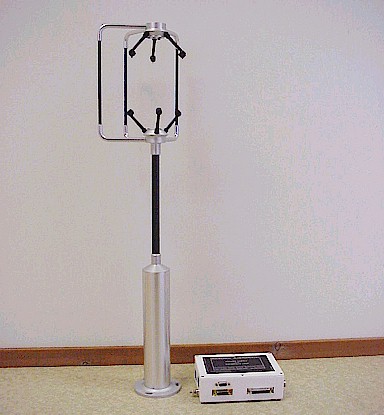 |

| Portable computer is registering measurements using suitable program for this purpose.  Edisol (version 0.39) is DOS program . Program doesn't run properly in Windows 95, so we have first to start computer to in MSDOS mode.  All measurements are saved to hard disk. In a week computer has new data about 160 Mb, in a year about 4,5 Gb (over 10 000 files). | 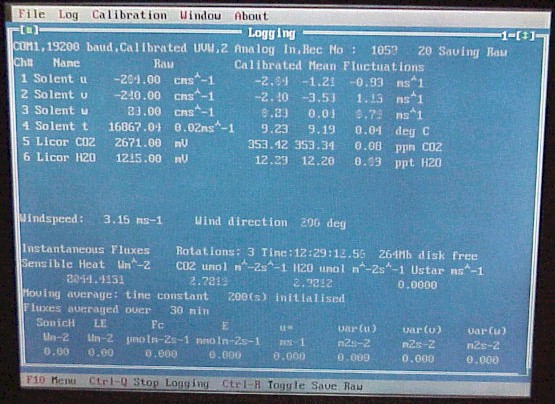 |
| --- | --- |

| Gas analyzer LI-6262 has to be calibrated from time to time and in Huhus we do that once a week.  First we let nitrogen flow in to analyzer. After ten minutes we adjust both water and carbon dioxide content reading to zero.  Secondly we put synthetic air (carbon dioxide 370 ppm) to inlet. After 5 minutes we adjust reading right.  For water calibration we have portable **dew point generator Licor LI-610**. We can put air to analyzer inlet from output of Licor LI-610. We adjust generator temperature. Setpoint is about two degrees colder than air temperature. After ten minutes we can adjust analyzer to show right reading. | 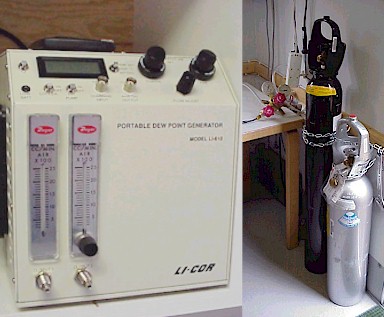 |
| --- | --- |

**Name of variables**

**2. Half-hour data documentation for meteorological factors (sheet ‘Meteorological factors’ in the file ‘DataHuhus2003 S1’) at Huhus site, in Finland in 2003**

# Principal Investigator/ Institutional Affiliation

Dr. Seppo Kellomäki

Faculty of Science and Forestry,

University of Eastern Finland, FIN-80101 Joensuu, Finland

e-mail: [seppo.kellomaki@uef.fi](mailto:seppo.kellomaki@uef.fi) Telephone: 0405032145

# Site Location and Description

The research was conducted in a 50-year-old stand of Scots pine (*Pinus* *syvestris* L.) at Huhus (62º52'N, 30º49'E, 145 m a.s.l.), eastern Finland. The stand density was 1176 trees ha^-1^ (ranging from 7.2 cm to 29.5 cm in diameter at breast height), with a mean height of 13.5 m above the ground and a mean diameter at breast height of 11.2 cm. The leaf area index was about 1.98 m^2^ m^-2^ ground area when flux measurements commenced. The soil is of a sandy podzol type. The top 50 cm contained an average volumetric mineral fraction of 47% and organic matter fraction of 21% and had a mean bulk density of 1.34 g cm^-3^. The climate is characterized by a long, cold winter. The mean monthly temperature is lowest in January, -10.4ºC, and highest in July, 15.8ºC. The average annual precipitation at the site (1961-2000) is 724 mm, of which 38% falls as snow. The understorey is principally mosses (*Dicranum spp, pleurozium schreberi*) and dwarf shrubs (*Vaccinium vitis-idaea, Calluna vulgaris*), so that the site represents the *Calluna* type, on a sandy soil with a low nitrogen supply.

The site is flat and there is a homogeneous underlying surface. The terrain is relatively level, extending at least 2 km around the tower used for the eddy covariance (EC) measurements. It is assumed that zero plane displacement ranges from 6.75 to 10.53 m and the roughness length is between 1.01 and 2.16 m. The fetch is about 460 m under unstable conditions and about 1890 m under neutral conditions.

# Methods for meteorological factors

Environmental and meteorological variables were measured using a Vaisala weather station (MILOS 500, Vaisala Oy, Helsinki, Finland) as placed at a distance of about 20 m from the eddy covariance mast. Temperature, humidity probes (HMP45D, Vaisala Oy, Helsinki, Finland) and anemometers (WAA15A and WAV15A, Vaisala Oy, Helsinki, Finland) were mounted at ~ 4, 9, 12, and 18 m above the ground along the weather mast to record the weather profile. Canopy temperature was measured with an infrared sensor (IR 4000.4GL, Everest Interscience, Inc. Tucson, USA) and net radiation above the canopy with a combination of an albedometer CM7B (Kipp & Zonen, Delft, Holland) and a CG2 pyrgeometer (Kipp & Zonen, Delft, Holland). Photosynthetically active radiation (PAR) above the canopy was measured with a quantum sensor (LI-190SA), and global radiation at a height of 20 m with a pyranometer (model CM6B/2, Kipp & Zonen, Delft, Holland). Precipitation above and below the forest canopy was measured using rain gauge (RG13, Vaisala Oy, Helsinki, Finland) and bole temperature with copper-constantan thermocouple probes inserted 1 cm into the trunk of each of three trees at heights of 15 cm, 285 cm, and 305 cm above the ground.

**Name of variables**

**3. Half-hour data documentation for meteorological factors (sheet ‘Soil heat flux’ in the file ‘DataHuhus2003 S1’) at Huhus site, in Finland in 2003**

# Principal Investigator/ Institutional Affiliation

Dr. Seppo Kellomäki

Faculty of Science and Forestry,

University of Eastern Finland, FIN-80101 Joensuu, Finland

e-mail: [seppo.kellomaki@uef.fi](mailto:seppo.kellomaki@uef.fi) Telephone: 0405032145

# Site Location and Description

The research was conducted in a 50-year-old stand of Scots pine (*Pinus* *syvestris* L.) at Huhus (62º52'N, 30º49'E, 145 m a.s.l.), eastern Finland. The stand density was 1176 trees ha^-1^ (ranging from 7.2 cm to 29.5 cm in diameter at breast height), with a mean height of 13.5 m above the ground and a mean diameter at breast height of 11.2 cm. The leaf area index was about 1.98 m^2^ m^-2^ ground area when flux measurements commenced. The soil is of a sandy podzol type. The top 50 cm contained an average volumetric mineral fraction of 47% and organic matter fraction of 21% and had a mean bulk density of 1.34 g cm^-3^. The climate is characterized by a long, cold winter. The mean monthly temperature is lowest in January, -10.4ºC, and highest in July, 15.8ºC. The average annual precipitation at the site (1961-2000) is 724 mm, of which 38% falls as snow. The understorey is principally mosses (*Dicranum spp, pleurozium schreberi*) and dwarf shrubs (*Vaccinium vitis-idaea, Calluna vulgaris*), so that the site represents the *Calluna* type, on a sandy soil with a low nitrogen supply.

The site is flat and there is a homogeneous underlying surface. The terrain is relatively level, extending at least 2 km around the tower used for the eddy covariance (EC) measurements. It is assumed that zero plane displacement ranges from 6.75 to 10.53 m and the roughness length is between 1.01 and 2.16 m. The fetch is about 460 m under unstable conditions and about 1890 m under neutral conditions.

# Methods for soil flux measurements

Soil heat flux (G) was determined with 4 soil heat flux plates (Radiation Energy Balance System, Seattle, WA, USA) buried 5 cm below the surface in a variety of microenvironments (ranging from mostly sunlit to mostly shaded). Soil temperatures at the depth of 2.5 cm and 3.5 cm were measured at four pits using thermalcouple sensors.

**Name of variables**
